# Effect of Organic Fertilizers on Selected Health Beneficial Bioactive Compounds and Aroma Profile of Red *Topepo* Sweet Pepper

**DOI:** 10.3390/foods9091323

**Published:** 2020-09-19

**Authors:** Adele Muscolo, Teresa Papalia, Carmelo Mallamaci, Sonia Carabetta, Rosa Di Sanzo, Mariateresa Russo

**Affiliations:** 1 Soil Chemistry and Soil Ecology Laboratory, Dipartimento di Agraria, University of Reggio Calabria, Via dell’Università, 25, 89124 Reggio Calabria, Italy; teresa.papalia@unirc.it (T.P.); carmelo.mallamaci@unirc.it (C.M.); 2 Food Chemistry, Safety and Sensoromic Laboratory (FoCuSS Lab), University of Reggio Calabria, Via dell’Università, 25, 89124 Reggio Calabria, Italy; sonia.carabetta@unirc.it (S.C.); rosa.disanzo@unirc.it (R.D.S.); mariateresa.russo@unirc.it (M.R.)

**Keywords:** antioxidants, aroma profiling, compost, phytochemicals, sweet pepper

## Abstract

Phytochemicals and antioxidant properties of red sweet pepper cv *Topepo* grown in soil amended with different organic fertilizers were compared with that grown in unamended soil. Organic fertilizers are an environmentally friendly alternative to recovery infertile soils that resulted from the intensified agricultural practices in red *Topepo* production. The aim was to discriminate the effects of organic fertilizers one from each other on the quality of red *Topepo* to find out the better sustainable fertilization practice for its cultivation. Results showed that compost from vegetable residues (CV) enhanced the synthesis of total phenols, flavonoids, ascorbic acid, vitamin E, carotenoids, anthocyanins, as well as carbohydrates, antioxidant activities, and aroma profiling, compared to horse manure (HD), compost from olive pomace (CO), and control (CTR). The results indicated a specificity between the quality of red *Topepo* and compost composition, highlighting that vegetable residues increased the synthesis of secondary metabolites, enhancing sustainably, the nutraceutical, sensorial, and economic value of red *Topepo*. The fertilizer composition resulted largely responsible for the synthesis of bioactive compounds, flavor, and aroma of this fruit.

## 1. Introduction

Pepper (*Capsicum annuum* L.) is nowadays one of the most popular vegetables worldwide, and in particular in the arid regions, with a production of 18 million tons per year [1]. Pepper is widely consumed for its culinary versatility and nutraceutical properties, both fresh and cooked, as, unlike other vegetables, contains few calories (100 g provide about 30 calories) and fat. Pepper is rich in vitamin C, polyphenols, chlorophylls, carotenoids, sugars, as well as flavonoids (lutein, zeaxanthin, and cryptoxanthin), considered potent antioxidants [2]. It is well demonstrated that the consumption of vegetables rich in bioactive compounds have beneficial effects on human health, protecting against the oxidative damage to cells and preventing the onset of degenerative diseases such as cancer, cardiovascular diseases, diabetes, Alzheimer’s, and Parkinson’s [2]. These bioactive compounds are also involved in the prevention of essential fat oxidation within the brain cells, helping to maintain an optimal brain function [3]. Bioactive compounds, mineral content, and organoleptic aspects are highly modulated by the growing conditions, and in particular, by the type of fertilization used [4]. The most used fertilizers for crop cultivation are chemical fertilizers because they cost less expensive and their nutrients are more quickly available to plants than organic fertilizers. However, long-term repeated use of chemical fertilizers may cause many negative environmental effects. An awareness in this field is therefore shifting the interest of farmers to organic cultivation systems which are becoming more in demand. Nowadays, there is increasing attention to organic or environmentally friendly agriculture and consumers are more oriented to healthier and more nutritious products. Regarding the quality of crops, Dumas et al. [5] demonstrated that treatment with chemical fertilizers can reduce the antioxidant levels in crops. Young et al. [6] and Vågen et al. [7] showed that vegetables such as cabbage, spinach, and pepper contained more antioxidants when cultivated with organic than chemical fertilizers. Akiyama et al. [8] showed that nutritional values of tomatoes grown with organic fertilizers were higher than those amended with chemical fertilizers. A specific study on sweet pepper showed that the addition of organic fertilizers improved its vegetative growth [9], total yield [10], and germination [9]. However, very limited information is available on how organic fertilizations affect sweet pepper quality, in terms of bioactive compounds and aromatic profile. The aims of the present study were: (1) to evaluate how different organic fertilizers (horse dung, HD; compost from wastes of traditional olive oil production systems, CO; compost from wastes of fourth range vegetables industry, CV) affected primary, secondary metabolites and antioxidant systems in fruits of red *Topepo*; (2) to investigate on, and compare to, the qualitative and quantitative changes in aroma-related volatile compounds, in red *Topepo* sweet pepper (RTSP) differently fertilized with the specific objective to identify the organic fertilizer more suitable to increase, in a sustainable way, its quality. 

## 2. Materials and Methods

### 2.1. Organic Fertilizers 

In this study, composts produced by olive pomace (CO) and vegetal residues (CV) that were extensively characterized in previous works [11,12] have been used. CO was rich in total organic carbon (49%), with a cation exchange capacity of 23.80 (cmol^(+)^ Kg^−1^) and a total nitrogen content of 1.89%, organic nitrogen/total nitrogen of 91.00% and water-soluble phenols of 2.31, mg tannic acid equivalent (TAE g L^−1^) d.w. CV was rich in macronutrients, with 24% of total organic carbon and a cation exchange capacity of 33.00 (cmol^(+)^ Kg^−1^), 2.03% of total nitrogen, organic nitrogen/total nitrogen ratio of 64%, and water soluble phenols of 7.03, mg TAE g L^−1^ d.w., [11]. HD had a cation exchange capacity of 18.80 (cmol^(+)^ Kg^−1^), 18% of total organic carbon, 0.89% of total nitrogen, 7.03, mg TAE g L^−1^d.w. of water soluble phenols, and an organic nitrogen/total nitrogen ratio of 68%.

### 2.2. Red Topepo Cultivation and Experimental Design 

Experimental sites were located in Motta San Giovanni, Loc. Liso, Italy (x:561023,1; y: 4204908,9; WGS 84 UTM Zone 33 N), the soil is a sandy-loam (11.85% clay, 23.21% silt, and 64.94% sand) textural class according to the Food and Agriculture Organization of the United Nations (FAO) soil classification system [13]. The soils were slightly alkaline and contained 3.09% organic matter and 0.17% nitrogen. Soil amendment was performed in triplicates in field, separated in parcel of 1 m square each. In each parcel, 3–4 plants/m²of red *Topepo* were transplanted exactly at the same size (six leaf stage). Composts and horse dung were added in each parcel on the basis of their own content of organic matter and precisely 1.5 q/ha for CO, 3.1 q/ha for CV, and 4.3 q/ha for HD. Unfertilized soil was used as control. Plants were watered regularly to maintain water content at 70% of field capacity in all the parcels. The experiment was arranged in a randomized complete block design and replicated three times. RTSP fruits of three replicates consisted of ~50 ripe fruits. The differently treated RTSP were collected at the same ripeness state on the basis of the visual characteristics (size, shape, and color). RTSP cultivated with CV matured in 88 days, with CO in 94 days, with HD in 95 days compared to control 100 days. The average fruit weight for each plant and for all the treatments was ~ 150 g. 

### 2.3. Sample Preparation

A portion of the differently treated RTSP fruit samples, harvested at the same state of ripeness, was stored at −20 °C until the preparation of the extracts. Before proceeding, red *Topepo* fruit was dried in a ventilated oven and ground using a mortar and pestle. 

The analyses of the volatile fraction were immediately carried out on freshly picked fruits cut into small pieces. The samples were not subjected to grinding to avoid the development of secondary compounds.

### 2.4. Preparation of Ethanol and Water Extracts

The extracts were obtained using the method described by Kang [14] with some modifications as reported in Muscolo et al. [15]. 

### 2.5. Total Soluble Proteins 

Soluble proteins were determined using the Bradford method as reported in Muscolo et al. [15]. Bovine serum albumin >99% purity (Sigma) was used and soluble proteins were estimated as mg/g FW.

### 2.6. Total Available Carbohydrates 

The total available carbohydrates were measured using the anthrone method with minor modifications as reported in Muscolo et al. [15]. 

### 2.7. Total Water Soluble Phenols, Ascorbic Acid, Total Carotenoids, Total Flavonoids, and Vitamin E 

Total water soluble phenols were measured using the Folin-Ciocalteu assay [16] with few changes as reported in Muscolo et al. [15]. 

For ascorbic acid determination, the red *Topepo* powder (0.10 g) was extracted with a solution of meta-phosphoric acid (3%)–acetic acid (7.98%), centrifuged at 2365× *g* (4000 rpm) for 10 min, and the supernatant was used for the determination of ascorbic acid using the method of Davies and Masten [17].

For vitamin E, the powder of red *Topepo* sweet peppers (0.10 g) was extracted with 10 mL of hexane:isopropanol solution (3:2 *v/v*), with agitation for 5 hr, and centrifuged at 1331× *g* (3000 rpm) for 10 min. The supernatant was used for the determination of vitamin E [18]. The absorbance was recorded at 695 nm. Quantification of vitamin E and of other reducing species was based on the molar absorption coefficient of the phosphor-molybdenum complex.

Flavonoids were estimated using the aluminium chloride colorimetric method of Djeridane et al. [19]. The absorbance was measured at 430 nm. Flavonoids were calculated from a calibration curve of rutin and expressed as rutin equivalents per gram of dry weight (RE/g dw).

For total carotenoids, the powder of red *Topepo* fruits (0.10 g) was extracted with 10 mL of hexane:isopropanol solution (3:2 *v/v*), with agitation, for 5 hr, centrifuged at 148× *g* (1000 rpm) for 10 min as reported by Zhang et al. [20]. The absorbance of the solution was measured at 450 nm and expressed as reported by Zhang et al. [20]. 

### 2.8. Mineral Assay

Cations (Na^+^, K^+^, Ca^++^, Mg^++^) were extracted from fruits of red *Topepo* and analyzed using ion chromatography (DIONEX ICS-1100, Thermo Fisher Scientific, Waltham, MA, USA) as reported in Muscolo et al. [15]. P was measured at 430 nm wavelength and comparing the results with the standard curve [21]. 

### 2.9. Antioxidant Activities 

The method of Blois [22] was used to determine the 2,2,1-diphenyl-1-picrylhydrazyl (DPPH) radical scavenging assay. The change in absorbance of the violet solution was measured at 517 nm after 30 min of incubation at 37 °C. DPPH activity was expressed as % of inhibition. The inhibition (%) of radical scavenging activity was calculated by the following equation:

(1) $$Inhibition\;\left(\%\right)=\frac{A_{0}-A_{s}}{A_{0}}\times 100$$

where *A_0_* is the absorbance of the control and *A_S_* is the absorbance of the sample after 30 min of incubation. 

Ferric reducing/antioxidant power (FRAP) assay was carried out using the method of Benzie and Strain [23] with minor modification as reported in Muscolo et al. [15]. 

The 2,2′-Azinobis-(3-Ethylbenzthiazolin-6-Sulfonic Acid) (ABTS) assay, also known as Trolox equivalent antioxidant capacity assay (TEAC) was done according to Pellegrini et al. [24] with few modifications as reported in Muscolo et al. [15]. 

Ferrozine-based colorimetric assay in terms of chelation ability of the ethanol extract was measured using the method of Dorman et al. [25] with few modifications as reported in Muscolo et al. [15]. 

### 2.10. Analysis of Volatile Compounds 

The headspace composition was investigated by headspace solid-phase micro-extraction (HS-SPME) coupled to gas chromatography (GC) separation and quadrupole time-of-flight mass spectrometry (QTOF/MS) detection. 

A DVB/CAR/PDMS (divinylbenzene/carboxene/polydimethylsiloxane) fiber was chosen [26,27,28,29], taking into account a recent study [30] that concerned the development of a SPME-GC/MS quantitative method to detect alkyl-2-methoxypyrazines (3-isopropyl-2-methoxypyrazine, 3-sec-butyl-2-methoxypyrazine, and 3-isobutyl-2-methoxypyrazine) a class of volatile organic compounds which are the impact compounds of the character of the genus *Capsicum* [31]. 

The extraction of volatile compounds was carried out with a 50/30 μm fiber DVB/CAR/PDMS (Supelco, Bellefonte, PA, USA) on aliquots of 3 g of cut samples with the addition of 0.5 g of NaCl, placed in a 20.0 mL SPME headspace vial. The headspace extraction step was carried out for 30 min at 60 °C and then the analytes were desorbed in splitless mode at 250 °C for 2 min. The fiber was then reconditioned for 10 min at 270 °C to ensure no carry-over of compounds from the previous sample. All of the samples were analyzed in triplicate and the results are mean values. The GC 7890A (Agilent Technologies, Inc., Santa Clara, CA, US) coupled into a QTOF 7200 accurate mass selective detector (Agilent Technologies, Inc., Santa Clara, US) was used to analyze the sample headspace components. Volatile compounds were separated by using a capillary column HP-5MS (crosslink 5% phenyl-methyl-siloxane, 30 cm *0.25 mm i.d., 0.25 um film thickness, Agilent Technologies, Inc., Santa Clara, CA, US). The oven temperature was held at 50 °C for 5 min and then increased at 4 °C/min to 180 °C for 3 min. The total run time was 52 min. The carrier gas was helium (purity > 99.999%) at a flow rate of 1.2 mL/min. The accurate mass scan spectra were recorded from 20–550 amu with the electron ionization (EI) source to 70 eV. The MS source temperature was set to 265 °C and a solvent delay of 1 min was selected. Nitrogen was used as the collision gas at 1.5 mL/min. MassHunter software B.08 was used for the control of equipment and data acquisition. MassHunter Qualitative Analysis and MassHunter Quantitative Analysis, (Agilent Technologies, Inc., Santa Clara, US) version B.08, were used for data analysis. The Unknown analysis software (version B.08 Agilent Technologies, Inc., Santa Clara, US), was used for compound identification by comparing mass spectra with NIST11 library data. 

### 2.11. Ultra-Fast Gas Chromatography Analysis

The ultra-fast gas chromatography (UFGC) analysis (mod. Heracles II, Alpha MOS, Toulouse, France) coupled with an Odorscanner headspace autosampler (mod. HS 100, CTC Analytics, Zwingen, Switzerland) to automate sampling and injection was used. The Heracles II was equipped with two metal columns of different polarities working in parallel mode: a non-polar column (MXT-5: 5% diphenyl, 95% methylpolysiloxane), and a mid-polar column (MXT-1701: 14% cyanopropylphenyl, 86% methylpolysiloxane), both 10 m long and 0.18 mm in diameter, coupled to two flame ionization detectors (FID1 and FID2). Therefore, two chromatograms are obtained simultaneously, allowing a well-defined identification of the chemical compounds. The instrument is operated through AlphaSoft 12.4, software that can be used within the additionally AroChemBase module (Alpha MOS, Toulouse, France). The data collected can be analyzed like that of a conventional chromatograph, using Kovats retention index (RI) values and Alpha MOS’ AroChemBase neural network to identify specific compounds. Every single compound, detected using the FID detector, can be identified by Alpha MOS’ AroChemBase software by comparing the retention indices of a sample’s peaks to those of known standards to give a list of compounds that could be responsible for each particular peak; the software calculates how different an unknown compound is from the nearest standard. A standard mixture of C6-C16 n-alkanes (from n-hexane to n-hexadecane) is used to calibrate the system, to allow retention time conversion into KI or RI. In addition to classical chromatography functionalities, the software for the instrument management and control provides chemometrics data processing tools to perform multivariate statistical analysis, such as sample fingerprint analysis and comparison, qualitative and quantitative models, quality control charts. 

The analytical parameters optimized for this analysis as follows: an aliquot of each sample (1.5 g) was placed in a 10 mL glass vial, sealed with magnetic plugs. The vials were placed in the Heracles’ auto sampler and left to equilibrate for approximately 20 min at 60 °C before beginning headspace analysis. Syringe pierced the silicone septum of the magnetic plug and for each sample, approximately, sampled 2 mL the headspace delivered at 125 µL/s by the autosampler to the injector at 200 °C. The 2 mL headspace aliquot was, before the chromatographic separation, adsorbed on a TENAX absorbent trap maintained at 50 °C for 20 s, while the carrier gas (H2) flowed (flow rate: 1 ml/min) through it in order to concentrate the analytes and to remove excess air and moisture.

Subsequently, desorption was obtained by increasing the temperature of the trap up to 250 °C in 93 s and the sample was injected. The thermal program started at 50 °C (held for 2 s) and increased up to 80 °C at 1 °C s^−1^; to 250 °C at 3 °C s^−1^ and held for 21 s. The total separation time was 110 s.

### 2.12. Statistical Analysis

Analysis of variance was carried out for all the datasets. One-way ANOVA with Tukey’s honestly significant difference test were carried out to analyze the effects of organic fertilizers on nutrients (proteins, carbohydrates, and minerals), bioactive compounds (vitamin E, total phenols, ascorbate, and flavonoid), and antioxidant capacity (ABTS, DPPH, FRAP, and ferrozine). SYSTAT 13.2 – Powerful Statistical Analysis and Graphics Software for Windows 7 was used for all the statistical analyses. Effects were significant at *p* ≤ 0.05.

The analysis of variance and multivariate analysis of aroma profiles were performed with SPSS statistical software program (version 20, IBM Statistics, New York, NY, US). 

Principle component analysis (PCA-XLSTAT software version 2015.5, Addinsoft, Paris, France) was performed both for the evaluation of volatile compounds datasets and to the data fusion approach (derive the first two principal components from the metabolic and antioxidant dataset and aroma profiles obtained by UFGC and GC-QTOF/MS analysis). Data management of all aroma compounds datasets was carried out using principal component analysis (PCA) on peak areas that were automatically calculated by the software that controls each instrument. 

## 3. Results and Discussion

### 3.1. Minerals, Primary and Secondary Metabolites of Red Topepo Fruits

Organic fertilizers, CV, CO, and HD induced the earlier ripening of the red *Topepo* fruits compared to the control and, among these, HD induced the earliest one. The amount of minerals (Table 1) in red *Topepo* fruits increased in samples grown with organic fertilizers compared to CTR (control), except for Na^+^. CV enhanced significantly calcium, potassium, phosphate, and sulfate contents in respect to the other treatments. Epidemiological and clinical studies showed the key role of calcium and potassium intake in regulating blood pressure [32], in reducing the risk of stroke, in preventing the development of renal vascular, glomerular, and tubular damage, [33,34] in decreasing urinary calcium excretion, and kidney stone formation [35]. Calcium and phosphate are also fundamental to human physiology (e.g., neuromuscular function) and are necessary to avoid skeletal demineralization (osteoporosis) [36]. Sulfur, after calcium and phosphorus, is the most important mineral with a key role as metabolic intermediates in plant metabolism [37]. The observed increase of the above mentioned nutrients confers to the RTSP fruits an additional nutritional value when grown with CV, as reported by de Jesús Ornelas-Paz et al. [38] and Gougoulias et al. [39]. Red *Topepo* sweet pepper contains essential nutrients and antioxidants (d) that can be modulated by external factors (climate, agricultural practices, stress). Our data (Table 2) confirm the previous findings, showing that the diverse organic fertilizations differently influenced red *Topepo* metabolism, leading to changes in the quantity and quality of its primary and secondary metabolites. Soluble protein increased in fruits of red *Topepo* grown in presence of organic fertilizers in respect to CTR and the greatest increase was observed in presence of HD. Total carbohydrates relevantly increased in organically RTSP in respect to CTR. The greatest increase was observed in fruits grown with CV, followed by those cultivated with CO, HD, and CTR. Regarding the secondary metabolites, the greatest increase in total water soluble phenols was observed in red *Topepo* in the following order CV > HD > CTR ≈ CO. 

Oloyede et al. [40], Sabrina et al. [41], Omar et al. [42], and Vignesh et al. [43] demonstrated that fertilization affected the level of secondary metabolites in plants, suggesting that the macronutrients, and in particular their concomitant presence in fertilizers, have a significant effect on the accumulation of phenols and antioxidants [44]. As demonstrated by Ibrahim et al. [45] in *Labisia pumila Benth var. alata*, the potassium fertilization levels increased not only the production of soluble proteins but also the carbohydrate content that simultaneously increased the synthesis of secondary metabolites in *L. pumila*, increasing the health promoting effects of this plant. Additionally, Nell et al. [46] evidenced in *Salvia officinalis* L., an increase in secondary metabolites in the presence of a high percentage of phosphorous. De Bona et al. [47] showed in cereal species, that increasing S fertilization enhanced the N and C uptake efficiency and use as well as the synthesis of plant bioactive compound because S is an essential constituent of key enzymes involved in the secondary metabolite synthesis [48].

Our results confirmed these findings and are in agreement with data of Toor and Savage [49] and Sereme et al. [50], providing evidence that the chemical composition of organic fertilizers modulates the synthesis of secondary metabolites in plants. CV richer in macronutrients than the other fertilizers stimulated plant metabolism and antioxidant compound synthesis more than the other two organic fertilizers. CV enhanced also significantly the amounts of ascorbic acid, vitamin E, doubling in particular the amount of carotenoids in respect to the other treatments and CTR.

Total water soluble phenols, ascorbic acid, flavonoids, vitamin E, and carotenoids are well known powerful antioxidants with a key role in the prevention of various chronic metabolic disorder including cancers and cardiovascular diseases, thus their observed increase, modulated by the use of organic fertilizers and particularly by CV, can be a way for increasing sustainably the economic and nutraceutical values of red *Topepo*. It was well demonstrated in the medical field that the regular use of fruits and vegetables with high contents of antioxidants raise the antioxidant capacity of serum/plasma [51]. The antioxidant compounds contained in fruit and vegetables are in fact able to inhibit Fe^3^ induced oxidation, to scavenge free radicals and also to work as reductants [52,53]. 

### 3.2. Antioxidant Activities

In this study, fruits of red *Topepo* treated with CV had the greatest level of antioxidants and also the highest scavenging activity (Table 3). Free radical scavenging is one of the known mechanisms by which antioxidants inhibit lipid oxidation caused by free radicals [54]. In this study, antioxidant capacity detected as ABTS and DPPH assays was more strongly correlated with total water soluble phenol and total flavonoids, which increased in all organic treated RTSP and moreover when grown with CV. Our data are in agreement with previous results of Chun et al. [55] and Kim et al. [56] on fresh plums showing that although the DPPH assay is not specific to any particular antioxidant components, the radical scavenging activity, through the hydrogen donation in red *Topepo* fruits, may be due to the hydroxyl groups contained in the antioxidant compounds. The order of DPPH-scavenging activities in the RTSP subject to the different treatments followed a similar trend. The highly significant relationship between DPPH and ABTS with total water soluble phenols and flavonoids, observed in this study, highlighted that phenolic compounds contributed relevantly to the antioxidant capacity of the studied crop species, in accordance to previous findings of Cai et al. [57] and Khodaie et al. [58] who found a linear correlation between the content of total water soluble phenolic compounds and antioxidant capacity of several plant species. Radical scavenging activity detected as FRAP seemed instead more due to the anthocyanin activity in organically treated fruits. Carotenoids have a protective role in numerous reactive oxygen species (ROS)-mediated disorders, such as, i.e., cardiovascular diseases, several types of cancer or neurological, as well as photosensitive or eye-related disorders. Compared to the single antioxidants, vitamins E, C, and β-carotene all together exhibit cooperative synergistic effects, resulting in a more effective scavenging activity than a single compound [59]. Carotenoids, acting in cooperation with two other individual antioxidants, vitamins E, and C, exhibited cooperative synergistic effects [60] with consequent increase in more than one antioxidant activity. Our data confirmed these findings evidencing that at an increase in carotenoids, ascorbic acid, and vitamin E corresponded also to an increase in FRAP and ferrozine activities.

### 3.3. Aroma Profiling

A total of 81 flavor related compounds, with an accurate mass matching above 80%, were identified. Table 4 shows the identified chemical classes: pyrazines (2), alcohols (12), aldehydes (12), esters (9), ketones (2), terpenoids (37) of which (19 sesquiterpens and 17 terpenes), hydrocarbons (7) and other compounds (1). 

**Figure 1:**
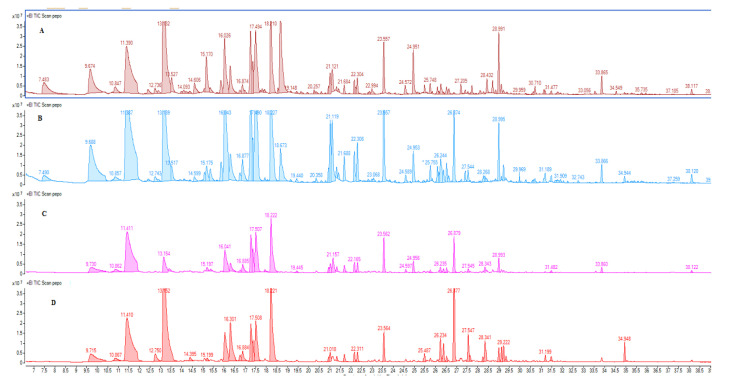
Chromatographic profiles total ion current (TIC) of red *Topepo* fruits obtained by cultivation: (**A**) without fertilizers (CTR); (**B**) horse dung (HD); (**C**) olive compost (CO); (**D**) vegetable compost (CV).

The chromatographic profiles (Figure 1) of different red *Topepo* fruits showed wide differences in the peak areas of the main compounds and the amount distributions were different for each sample.

Compared to CTR and to CV, the CO, treated red *Topepo* fruits displayed a lower chromatographic complexity correlated to a lower intensity of the volatile fraction. In CTR, CO, and CV treated red *Topepo* fruits, terpenoids (monoterpenes and sesquiterpenes) were the most abundant while in HD-grown RTSP, a similar concentration of alcohols (most representative family), terpenoids, and aldehydes was found. Despite the high number of detected volatile compounds, only some of them can be considered useful in the classification of red *Topepo* aroma. In particular, between the identified compounds, 2-isobutyl-3-methoxypyrazine (green bell pepper), 2-sec-butyl-3-methoxypyrazine (carrot, lettuce, grassy), hexanal (grassy), nonenal (cucumber, herbal), 3-carene (red bell pepper, rubbery), β-ocimene (rancid, sweaty), (E) 4-nonanone, (E,Z) 2,6 nonadienal, (E,E) 2,4 decadienal are responsible for the green-related odor notes [61] and are the compounds identified at different extent in the different samples. The different concentrations drove the differences in the aromatic fingerprintings.

Methoxypyrazines, heterocyclic aromatic organic compounds naturally present in green plant tissue (in particular 2-isobutyl-3-methoxypyrazine and 2-sec-butyl-3-methoxypyrazine) were first identified in green capsicum (bell pepper), where they were found to be the principal compound responsible for the distinct capsicum odor. 2-isobutyl-3-methoxypyrazine, also known as *Grindstaff pyrazine*, confers to the fresh green bell peppers their distinctive smell and for this, is just called *bell pepper pyrazine* [62,63,64]. In short, both methoxypyrazines and 2-isobutyl-3-methoxypyrazine can be considered quality markers of aromatic profile of our samples.

Analysis of each profile and peak area showed that apart from the pyrazines, in all the samples analyzed, terpenes were the main compounds with large intra-class variations. In red *Topepo* sweet pepper fruits treated with CV, the highest concentrations of pyrazines and terpenoids were found. Terpenoids are of great importance in determining the aromatic quality of vegetables and have a broad range of biological properties including cancer chemo preventive effects, antimicrobial, antifungal, antiviral, anti-hyperglycemic, anti-inflammatory, and anti-parasitic activity [65]. The aromatic fraction of sweet pepper fruits fertilized with CV was characterized both by the highest percentage of pyrazines and the highest concentration of terpenes, followed by esters and alcohols. A similar trend, although with lower percentages of terpenes in favor of aldehydes, was found in sweet pepper fruits grown with CO.

The results are consistent with the type of compost used. In fact, CO obtained from olive pomace was less rich in macronutrients than CV. In CTR, a greater presence of aldehydes emerged, while in HD, a chemical class of compounds did not prevail, rather, a similar and decreasing tendency among terpenes, esters, aldehydes, and finally, alcohols was observed. Among terpenes, CV-grown sweet pepper fruits showed the lowest presence of sesquiterpenes compared to the other samples, suggesting their poor contribution to the aromatic intensity of the vegetables. The characterizing terpene was 3-carene, which represents almost half of the entire class, followed by *trans*-caren-3-ol while, among the sesquiterpenes, β-curcumene is the compound that most contributed to the aromatic profile, followed by a contained presence of α-santalene.

The CO samples contained, albeit very small, both 3-carene and *trans*-caren-3-ol and an intra-class inversion in favor of sesquiterpenes, which is the most consistent and entirely represented by α-santalene followed by β-curcumene.

A similar pattern was observed in the control and in HD treated red *Topepo* fruits in which the main compounds were 3-carene and the *trans*-caren-3-ol, although in lower concentrations compared to CV. Among the sesquiterpenes, α-copaene and β-curcumene were detected. Aldehydes (E, Z)-2,6-nonadienal, (Z)-2-nonenal, and 7,11-hexadecadienal were also present in all samples. CO had the highest quantity of hexanal, while CTR contained high quantity of *cis*-decenal. Among the esters, 4-hexen-1-ol acetate was detected, exclusively, in the CTR and HD treated samples. 4-methyl-1-pentanol was the main alcohol.

**Figure 2:**
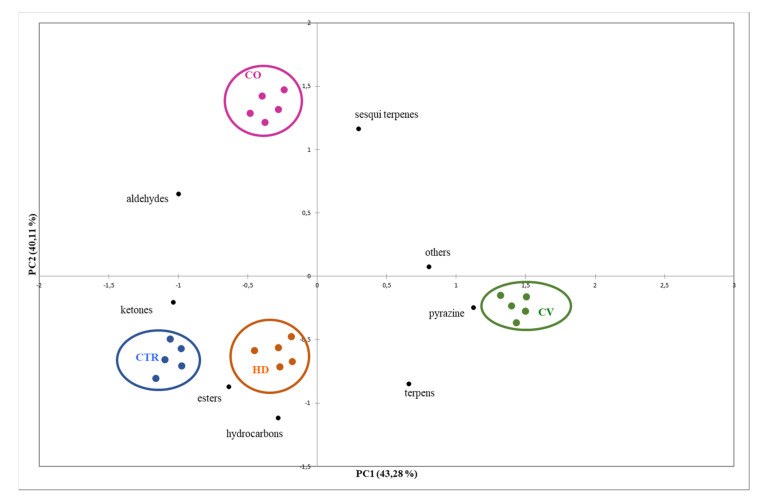
Bi-plots PCA (principal component analysis) of SPME-GC-QTOF/MS (solid-phase micro-extraction coupled to gas chromatography separation and quadrupole time-of-flight mass spectrometry detection) dataset.

Principal component analysis (PCA) confirmed the above results (Figure 2). The changes in volatile fraction composition of the samples due to the organic fertilizers used gave rise to chemical compounds and different chromatographic profiles that were used for the purpose of classification using chemometrics.

The screen plot of the variance captured by each PC, shows that the total variability of the data can be concentrated in just few new coordinates (Figure 2). The first two principal components captured 83.39% of the whole data variance. The first PC alone is enough to separate three groups (CTR, HD, and CV), while the second PC allows the discrimination of CO samples.

A detailed evaluation of the chromatographic peaks allowed to establish the relationship between PCs and chemical classes, and more specifically, the distribution of the samples in the PC space revealed the high contribution of pyrazines and terpenens.

**Figure 3:**
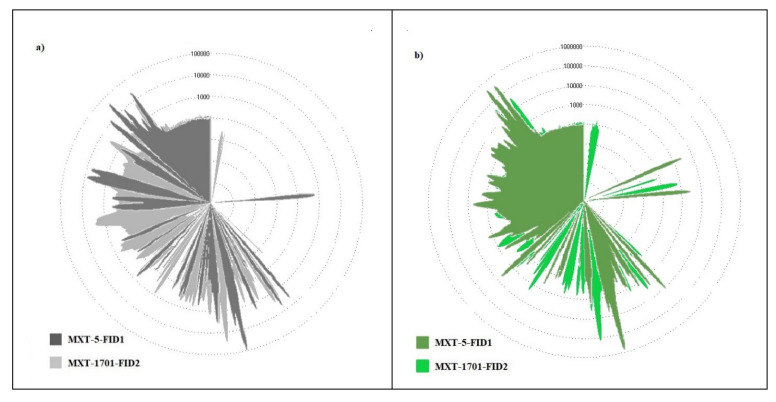

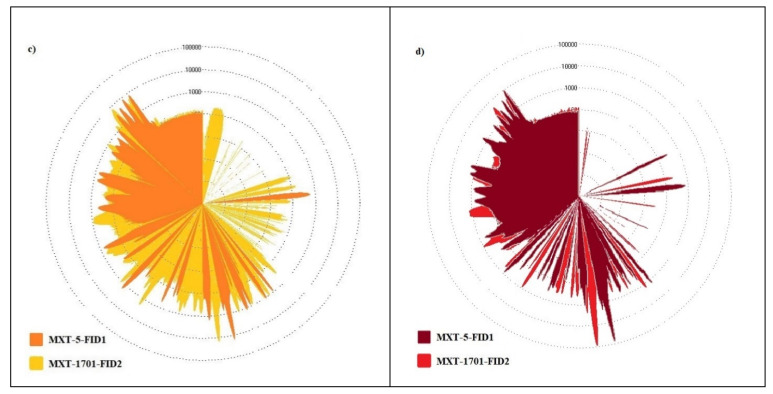
Radar plots (odor maps or fingerprints), obtained by using an electronic nose with MXT-5 column and MXT-1701 column, of red *Topepo* fruits cultivated (**a**) without fertilizers (CTR); (**b**) with vegetable compost (CV); (**c**) with olive compost (CO); (**d**) with horse dung (HD).

The processing of the UFGC results using the AroChemBase software allowed the identification of thirty volatile compounds while the odor maps of the red *Topepo* fruits, representing the “Odor fingerprints” (Figure 3), were extrapolated from the chromatographic profiles.

The comparison of the Heracles II odor profiles (Figure 3) showed, clearly, the differences between the samples treated with different organic fertilizers and between these and control.

According to SPME-GC-QTOF/MS results, 2-isobutyl-3-methoxypyrazine was identified in all the analyzed samples and it was present in the greatest amount in CV treated red *Topepo*. In all the samples, between alcohols and aldehydes, a great amount of heptanal, 2-nonenal, and 1- octen-3-ol compounds which give flavor notes of freshly cut grass or ground leaves, and 2-methyl-propanal, which gives the typical cacao spicy, were identified [64]. Myrtenol, citronellol, thymol, β-farnesene, α-phellandrene, and linalool, among the terpenes, were also pointed out.

The UFGC profiles, in the form of a numerical relationship between the retention time and the area of the peaks, were analyzed by using PCA. To ensure proper description of the real input data, it was assumed that the first two components described more than 80% of input set variance [66]. In order to reduce the dataset measurements consisting of all the peak areas of each analyzed chromatograms, the most discriminant peak areas of specific compounds were extrapolated ((Z)-2-nonenal, pentan-2-ol 2-methyl propanal) and then treated as an input dataset for PCA analysis [67].

**Figure 4:**
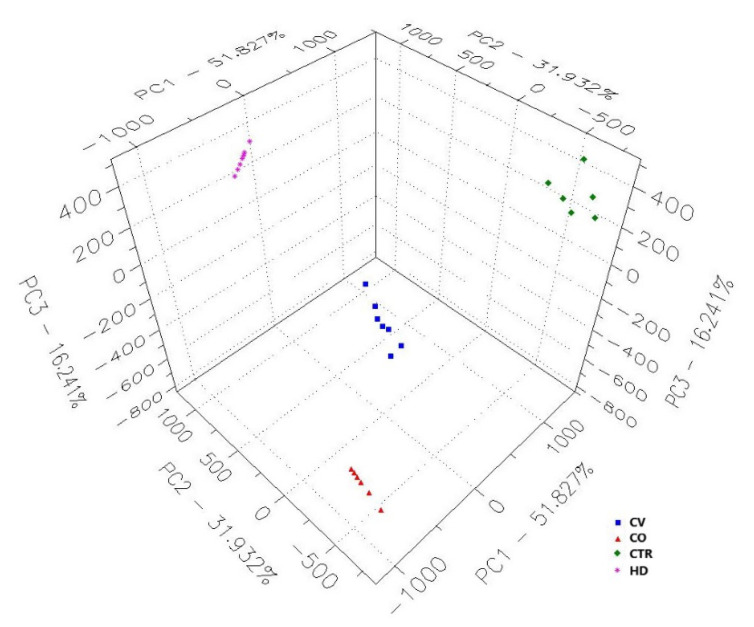
3D-PCA representation based on the volatile profiles of the different red *Topepo* sweet peppers obtained by UFGC (CV: vegetable compost; CO: olive compost; CTR: control; HD: horse dung)

Multidimensional comparative analysis PCA allowed the grouping of samples according to similarity, giving a discrimination index of 87%. The overall outcomes of the UFGC analysis are shown in the 3D-PCA graph (Figure 4). PCA showed a clear separation between all samples obtained with organic treatments (CO, CV, and HD) and CTR. The principal components PC1, PC2, and PC3 represented 51.82%, 31.93%, and 16.24% of the total variance, respectively. The cumulative contribution rate of the first two components accounted nearly 84%, which represented the largest fraction of overall variability in the observations.

### 3.4. Data Fusion

**Figure 5:**
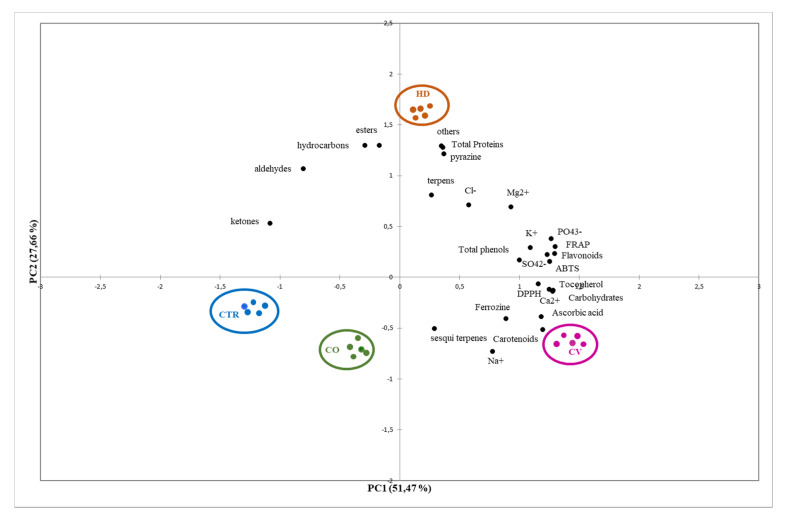
PCA Bi-plot for data fusion of red *Topepo* analysis.

The possibility of integrating the information present in the different dataset of each individual model allows an improvement in the discrimination between the differently treated samples. Therefore, a strategy of merging all the analytical data obtained was implemented. The data fusion strategy consisted of concatenating the original dataset so as to be able to analyze the resulting array as if it were a single data block. The results of the metabolic profile (concentration of primary and secondary metabolites and ions), antioxidant activity, chemical classes of the volatile fraction obtained by SPME-GC-QTOF/MS, were concatenated in a single array and the individual data normalized. Principal components analysis (PCA) biplot visualization (Figure 5) summarized the overall relations between the all variables in all samples.

The total variation was 79.13% (PC1: 51.47% and PC2: 27.66%). PCA showed a clear separation between all samples, indeed it showed that CV is clearly discriminated from the others as well as HD, confirming the quantitative and qualitative differences of red *Topepo* differently fertilized.

## 4. Conclusions

The application of organic fertilizers in various forms to the crops has so far been recommended to compensate for the lack of nutrients in soils, now, with this study, fertilizers from compost of organic wastes can be used instead to improve vegetable quality. Although the fruits of the treated red *Topepo* showed similarities in their shapes and sizes, their aromatic profiles, in good agreement with the secondary metabolites, have been strongly modified both in intensity and in composition by the different organic fertilizers that have differently influenced the production of bioactive compounds, increasing ascorbic acid, vitamin E, carotenoids, soluble phenols, and other phytochemicals. Taken together, these results highlight that the fertilization with compost produced on farm can be used to strengthen bioactive compounds in fruits, providing a new strategy to improve the nutraceutical potential and economic values of crops with important consequences on the bio and green economy.

## Figures and Tables

**Table 1:** Ion content (mg g^−1^ dw) in red *Topepo* fruits grown in unfertilized soil (control, CTR) and soils fertilized with compost from olive wastes (CO), compost from vegetable wastes (CV), and horse dung (HD).

Treatments	CTR	CO	CV	HD
Na^+^	1.25 ^b^ ± 0.05	0.94 ^c^ ± 0.05	1.80 ^a^ ± 0.10	0.85 ^c^ ± 0.06
K^+^	17.99 ^b^ ± 4.27	21.34 ^b^ ± 0.14	25.21 ^a^ ± 1.01	23.83 ^a^ ± 1.23
Ca^2+^	1.78 ^c^ ± 0.04	2.00 ^b^ ± 0.08	2.29 ^a^ ± 0.07	2.03 ^b^ ± 0.08
Mg^2+^	1.31 ^c^ ± 0.10	2.06 ^b^ ± 0.06	2.16 ^b^ ± 0.06	2.48 ^a^ ± 0.07
Cl^-^	1.14 ^d^ ± 0.01	2.25 ^b^ ± 0.09	1.86 ^c^ ± 0.03	2.55 ^a^ ± 0.05
SO_4_^2-^	2.40 ^d^ ± 0.27	4.32 ^c^ ± 0.11	6.44 ^a^ ± 0.15	5.39 ^b^ ± 0.13
PO_4_^3-^	2.62 ^d^ ± 0.12	3.65 ^c^ ± 0.09	4.86 ^a^ ± 0.06	4.49 ^b^ ± 0.14

Letters show significant differences among the treatments (Tukey’s test. *p*-level ≤ 0. 05).

**Table 2:** Primary and secondary metabolites in red *Topepo* fruits grown in unfertilized soil (control, CTR) and soils fertilized with compost from olive wastes (CO), compost from vegetable wastes (CV), and horse dung (HD). Carbohydrate, soluble protein, ascorbic acid, carotenoids, and vitamin E were expressed as mg/100 g FW. Total phenols were expressed as mg gallic acid/100 g FW and flavonoids as rutin (R) E/g FW.

ID	CTR	CO	CV	HD
Carbohydrate	0.64 ^c^ ± 0.11	1.20 ^b^ ± 0.03	1.61 ^a^ ± 0.11	1.18 ^b^ ± 0.21
Soluble protein	631.29 ^c^ ± 10.88	644.22 ^bc^ ± 30.11	707.48 ^b^ ± 44.42	1028.06 ^a^ ± 30.08
Total phenols	451.26 ^bc^ ± 53.51	405.57 ^c^ ± 15.51	643.22 ^a^ ± 58.05	519.46 ^b^ ± 16.27
Ascorbic acid	93.18 ^b^ ± 8.99	94.90 ^b^ ± 15.13	159.11 ^a^ ± 9.50	97.19 ^b^ ± 5.73
Carotenoids	614.67 ^c^ ± 13.32	637.97 ^c^ ± 10.03	1582.05 ^a^ ± 52.05	686.08 ^b^ ± 12.01
Vitamin E	0.63 ^b^ ± 0.01	0.69 ^b^ ± 0.01	1.05 ^a^ ± 0.18	0.90 ^a^ ± 0.01
Flavonoids	2.53 ^d^ ± 0.06	4.31 ^c^ ± 0.14	5.21 ^a^ ± 0.12	4.72 ^b^ ± 0.07

Letters show significant differences among the treatments (Tukey’s test. *p*-level ≤ 0. 05).

**Table 3:** Antioxidant activities (2,2-diphenyl-1-picrylhydrazyl radical (DPPH, % inhibition); ferric reducing antioxidant power (FRAP, (µM TE g^-1^ FW); Trolox equivalent antioxidant capacity assay TEAC ABTS %; Ferrozine (% inhibition) in red *Topepo* fruits grown in unfertilized soil (control, CTR) and soils fertilized with compost from olive wastes (CO), compost from vegetable wastes (CV), and horse dung (HD).

ID	CTR	CO	CV	HD
DPPH	60.67 ^b^ ± 7.02	70.67 ^b^ ± 11.46	93.33 ^a^ ± 5.86	84.33 ^a^ ± 5.15
FRAP	606.17 ^c^ ± 52.25	653.42 ^c^ ± 75.55	1304.84 ^a^ ± 107.97	809.49 ^b^ ± 168.74
ABTS	49.33 ^c^ ± 2.08	49.67 ^c^ ± 4.51	77.33 ^a^ ± 2.15	63.3 ^b^ ± 1.10
FERROZINE	25.00 ^b^ ± 2.00	24.00 ^d^ ± 1.00	57.00 ^a^ ± 3.00	14.00 ^c^ ± 1.00

Letters show significant differences among the treatments (Tukey’s test. *p*-level ≤ 0. 05).

**Table 4:** - Summary of compounds identified in red *Topepo* fruits grown in unfertilized soil control (CTR) and soils fertilized with compost from olive wastes (CO), compost from vegetable wastes (CV), and horse dung (HD).

COMPOUNDS	RT	CTR	CV	CO	HD
**ALCOHOL (12)**					
1-pentanol, 4-methyl-	6.11	x	x	x	x
guaiacol	14.62	nd	x	nd	x
cis- 3 nonen- 1-ol	15.35	x	x	x	x
phenylethyl alcohol	15.65	nd	nd	nd	x
benzyl alcohol	17.36	x	x	x	x
1.10-decanediol	19.15	x	nd	x	x
nona-3.5-dien-2-ol	20.11	x	nd	x	nd
4-tert buthylthiophenol	21.33	x	x	x	x
1-decanol. 2-ethyl-	22.43	x	x	x	x
1-octanol. 2-butyl-	23.13	x	x	x	x
1.2-benzenediol. O-(4-ethylbenzoyl)-O’-propargyloxycarbonyl-	23.55	nd	x	x	x
1-octanol. 3-butyl-	31.77	x	x	x	x
3-hexadecanol	37.27	nd	x	x	nd
**ALDEHYDES (12)**					
hexanal	4.08	x	x	x	x
benzeneacetaldehyde	12.91	x	nd	x	x
2-octenal. (E)-	13.51	nd	nd	x	x
4-nonenal. (E)-	14.96	x	nd	nd	x
2.6-nonadienal. (E.Z)-	17.27	nd	x	x	x
2-nonenal. (Z)-	17.49	x	x	x	x
Benzaldehyde. 4-ethyl-	18.05	nd	nd	nd	x
cis-decenal	18.68	x	nd	x	x
cuminaldeide	18.90	x	x	x	nd
7.11-hexadecadienal	21.14	x	x	x	x
2.4-decadienal. (E.E)-	22.24	x	nd	nd	nd
tricyclo[7.1.0.0[1.3]]decane-2-carbaldehyde	23.00	x	x	x	x
**KETONES (2)**					
4-nonanone	14.11	x	x	x	x
2(3H)-furanone. dihydro-5-pentyl-	24.59	x	x	x	x
**ESTERS (9)**					
4-hexen-1-ol. acetate	5.65	x	nd	nd	x
phenacylidene diacetate	9.69	x	x	x	x
cinammilcarbonilate	14.86	x	x	nd	x
methyl phenylacetate	16.99	x	nd	nd	nd
myrtenyl acetate	23.80	x	x	x	x
hexanoic acid. hexyl ester	25.33	x	nd	nd	nd
5.8.11.14.17-eicosapentaenoic acid. methyl ester. (all-Z)-	32.73	x	nd	x	x
oxalic acid. allyl octadecyl ester	34.55	x	nd	x	x
isopropyl myristate	37.90	nd	x	nd	nd
**HYDROCARBONS (7)**					
1.3-cyclopentadiene. 5.5-dimethyl-2-ethyl-	10.85	x	x	x	xx
1.4-cyclohexadiene. 3-ethenyl-1.2-dimethyl-	16.03	x	x	x	x
naphthalene. 2-methyl-	22.16	x	x	x	x
cyclododecane	25.48	nd	x	x	x
nonane	30.93	x	x	x	x
1-octadecyne	34.95	nd	x	x	x
trans-1.2-diphenylcyclobutane	35.86	nd	nd	x	x
**PYRAZINE (2)**					
pyrazine. 2-methoxy-3-(1-methylpropyl) (2-sec-butyl-3-methoxypyrazine)	17.92	x	x	x	x
pyrazine. 2-methoxy-3-(2-methylpropyl) (2-isobutyl-3-methoxypyrazine)	18.21	x	x	x	x
**TERPENS (17)**					
citronellal	12.41	x	nd	x	x
β-ocimene	12.75	x	x	x	x
3-Carene	13.18	x	x	x	x
p-cymene	14.39	x	x	x	x
carvacrol	15.08	x	x	x	x
(+)-4-carene	15.19	nd	x	x	x
cosmene	15.85	x	x	x	x
trans caren 3-ol	16.31	x	x	x	x
neo-allo ocimene	16.75	x	x	x	x
β-ciclocitral	19.65	x	nd	x	x
perillol	20.66	x	x	nd	x
cis-verbenol	21.46	x	nd	nd	x
thymol	21.69	x	x	x	x
(-)-myrtenol	22.82	x	x	x	x
geranil linalol	28.99	x	x	x	x
geraniol	30.66	x	x	nd	x
trans geranil geraniolo	31.20	nd	x	X	x
**SESQUI TERPENES (19)**					
(E)2, (Z)4, (E)6-allofarnesene	20.41	x	x	X	x
α-copaene	24.96	x	x	X	x
β-panasinsene	25.43	x	nd	nd	x
α-longipinene	26.23	x	x	X	x
α-santalene	26.38	x	x	X	x
β-curcumene	26.88	x	x	X	x
caryophyllene	27.17	x	x	nd	nd
ylangene	27.23	x	x	X	x
β-copaene	27.55	x	x	X	x
santene	27.64	x	x	X	x
di-epi-α-cedrene	27.83	nd	x	nd	nd
α-cubebene	28.15	x	nd	nd	x
nuciferol	28.25	x	x	X	x
cuparene	28.34	x	x	X	x
(e)-β-famesene	28.39	x	nd	X	x
cedrene	28.72	x	x	X	nd
α-himachalene	29.12	x	x	X	x
γ-murolene	29.57	x	x	nd	x
α-ylangene	30.14	x	x	nd	nd
**OTHERS (1)**					
2 methyl furan	16.87	x	x	X	x
